# Latitudinal Adaptation and Genetic Insights Into the Origins of *Cannabis sativa* L.

**DOI:** 10.3389/fpls.2018.01876

**Published:** 2018-12-21

**Authors:** Qingying Zhang, Xuan Chen, Hongyan Guo, Luisa M. Trindade, Elma M. J. Salentijn, Rong Guo, Mengbi Guo, Yanping Xu, Ming Yang

**Affiliations:** ^1^Industrial Crops Research Institute, Yunnan Academy of Agricultural Sciences, Kunming, China; ^2^Wageningen University and Research Plant Breeding, Wageningen University and Research, Wageningen, Netherlands

**Keywords:** Cannabaceae, industrial hemp, genetic diversity, phylogeography, cpDNA

## Abstract

Cannabis is one of the most important industrial crops distributed worldwide. However, the phylogeographic structure and domestication knowledge of this crop remains poorly understood. In this study, sequence variations of five chloroplast DNA (cpDNA) regions were investigated to address these questions. For the 645 individuals from 52 *Cannabis* accessions sampled (25 wild populations and 27 domesticated populations or cultivars), three haplogroups (Haplogroup H, M, L) were identified and these lineages exhibited distinct high-middle-low latitudinal gradients distribution pattern. This pattern can most likely be explained as a consequence of climatic heterogeneity and geographical isolation. Therefore, we examined the correlations between genetic distances and geographical distances, and tested whether the climatic factors are correlated with the cpDNA haplogroup frequencies of populations. The “isolation-by-distance” models were detected for the phylogeographic structure, and the day-length was found to be the most important factor (among 20 BioClim factors) that influenced the population structures. Considering the distinctive phylogeographic structures and no reproductive isolation among members of these lineages, we recommend that *Cannabis* be recognized as a monotypic genus typified by *Cannabis sativa* L., containing three subspecies: subsp. *sativa*, subsp. *Indica*, and subsp. *ruderalis*. Within each haplogroup which possesses a relatively independent distribution region, the wild and domesticated populations shared the most common haplotypes, indicating that there are multiregional origins for the domesticated crop. Contrast to the prevalent Central-Asia-Origin hypothesis of *C. saltiva*, molecular evidence reveals for the first time that the low latitude haplogroup (Haplogroup L) is the earliest divergent lineage, implying that *Cannabis* is probably originated in low latitude region.

## Introduction

Cannabis is one of the oldest crops and has been distributed worldwide by humans. This plant may have been utilized for at least 10,000 years (Schultes et al., 1974; Long et al., 2016), and its cultivation in China can be traced back to around 6,000 years ago according to the archaeological findings and records of ancient literatures (Li, 1974; Yang, 1991). Cannabis has been developed as a multi-purpose crop, which is widely used for the production of biomaterials such as textile, paper, construction, and insulation materials, but also as functional foods, namely the oil and seeds, and for other applications including cosmetics and personal care products, and in the pharmaceutical industry. The global market for hemp has been estimated to consist of more than 25,000 products (Johnson, 2013; Salentijn et al., 2015). In recent years, the hemp industry has increasingly received attention and the development of high value products has been the main focus of various studies (Amaducci et al., 2015). Especially, cannabis plants can produce more than 100 pharmacologically active compounds (cannabinoids), with the most studied compounds being tetrahydrocannabinol (THC) and cannabidiol (CBD), and CBD has sparked an increasing interest for product development.

Based on the content of cannabinoids in this herbaceous annual crop, cannabis plants have been often classified as hemp, mostly referring to a fiber crop with low tetrahydrocannabinol (THC) and marijuana, the drug type with often high THC content. This plant comprises both wild and domesticated populations which can be either dioecious or monoecious cultivars. The flowering is very sensitive to photoperiod and cultivars can be early-, intermediate-, and late-ripening. Compared to the domesticated cannabis, the wild forms usually exhibit the following distinct morphological and physiological features: remarkably smaller seeds (mature achene, thousand seed weight < 10 g), easy seed shattering behavior (seeds readily disarticulate from the pedicel), long-term seed dormancy and the need for cold-moist stratification treatment to facilitate germination. For a long time, researchers have disputed the taxonomy of *Cannabis* regarding the definitions of species, subspecies, and/or varieties (McPartland and Guy, 2004, 2014; Hillig, 2005; Gilmore et al., 2007; Small, 2015). The issue of *Cannabis* taxonomy continues to puzzle botanical taxonomists (Piomelli and Russo, 2016; Welling et al., 2016; Mcpartland and Guy, 2017; Mcpartland and Hegman, 2018). Linnaeus named *Cannabis sativa* L. (hereafter as *C. sativa*) as a unique species. Later on, two species, *C. indica* Lam. (1785) and *C. ruderalis* Jan. (1924), were split from *C. sativa* based on certain distinct morphological Characteristics (Hillig and Mahlberg, 2004), while Small and coauthors recommended retaining only one species (*C. sativa*) but including two subspecies, subsp. *sativa* and subsp. *indica*, where each subspecies includes both domesticated and wild varieties (Small and Cronquist, 1976; Small, 2015). Recently, based on allozyme analysis results, Hillig (2005) suggested a taxonomic concept of three species (*C. sativa, C. indica*, and *C. ruderalis*) including seven putative taxa in the genus *Cannabis*.

Germplasm collections of *Cannabis* are the most valuable fundamental materials for breeding as they are a potential source of novel genes controlling important traits such as increased seed productivity, improved qualitative characteristics for example fiber quality, or resistance to adverse environmental factors such as cold, drought, strong wind, and pest/disease pressure. The native distribution range of *Cannabis* is commonly believed to be in Central Asia, Siberia, the Himalayas, and possibly extending into China (de Candolle, 1885; Vavilov, 1926; Li, 1974; Hillig, 2005; Small, 2015; Mcpartland and Hegman, 2018). Currently, the distribution of cannabis covers most of the Chinese territory, ranging from about 23 to 51° N, 80 to 125° E. China has been a major hemp growing country with the largest cultivation area and has developed many landraces and cultivars. China is part of the potential center of origin for cannabis, with abundant genetic resources in wild populations but also developed cultivars, thus provides a unique opportunity to investigate the domestication origin of cannabis plants. However, the wild populations of cannabis have been poorly studied, and the genetic diversity and structure of these populations, as well as the relationships among the wild populations and the domesticated cultivars remains largely unknown.

Chloroplast DNA (cpDNA) markers and phylogeographic methods have been proven to be very useful tools in investigating genetic diversity, population structure, domestication origin, and historical context of species (Avise, 2000, 2004, 2009). The cpDNA is a haploid (and thus are homoplasmic), non-recombining genome that is maternally inherited in most angiosperms (Schaal et al., 1998; Avise, 2009). However, like many other plant species, cannabis cpDNA displayed very low genetic diversity (Gilmore et al., 2007; Zhang et al., 2017; Mcpartland and Hegman, 2018). A key to successful utilization of cpDNA markers for estimating diversity and phylogenetic relationships among populations of *Cannabis* species requires obtaining sufficient genetic variation in cpDNA and developing suitable cpDNA markers. In this study, based on scrutinizing differences in the whole chloroplast genomes DNA sequences of four *Cannabis* accessions (Oh et al., 2016; Vergara et al., 2016), we developed five DNA markers for the most variable polymorphic regions and investigated the genetic diversity of an extensive set of *Cannabis* samples. These samples include wild populations, representative landraces and breeding cultivars from China, as well as some accessions from other countries (The Netherlands, France, Hungary, Italy, Russia, Nigeria, Korea, and USA). Our main objectives were: (1) to estimate the genetic diversity and elucidate the distribution patterns of the wild and domesticated cannabis from China; (2) to determine the main factors that affected the spatial distribution of cannabis and provide information on historical processes of this plant; (3) to infer the genetic relationships between the populations or lineages, as well as domestication origins of cannabis cultivars in China.

## Materials and Methods

### Plant Material

The studied material comprised 645 *Cannabis* individuals (derived from 52 accessions: 25 wild populations and 27 domesticated populations or cultivars), and four closely related out group species, *Humulus scandens, Humulus yunnanensis, Humulus lupulus*, and *Aphananthe aspera*. Information relevant to the samples is shown in Table 1.

**Table 1:** Sample information and summary of haplotype distribution, genetic diversity for each population based on the combined five cpDNA regions.

**Code/Name**	**Origin/location**	**Type**	**No**.	**Latitude (^**°**^N)**	**Haplotypes (*Nh*)**	***Hd***	**π (×10^**−2**^)**
EG	Inner Mongolia, China	W	20	50.21	H1(19), H2(1)	0.100 ± 0.088	0.025 ± 0.020
HE	Inner Mongolia, China	W	27	49.28	H3(17), H6(10)	0.484 ± 0.054	0.013 ± 0.013
YK	Inner Mongolia, China	W	20	49.25	H3(19), H4(1)	0.100 ± 0.088	0.003 ± 0.006
JL	Jilin, China	W	13	45.02	H3(9), H4(4)	0.462 ± 0.110	0.013 ± 0.014
AL	Xinjiang, China	W	20	48.20	H1(20)	0.000	0.000
HG	Xinjiang, China	W	20	44.21	H1(13), H9(7)	0.479 ± 0.072	0.357 ± 0.188
YN	Xinjiang, China	W	24	43.84	H1(10), H9(14)	0.507 ± 0.045	0.379 ± 0.196
KS	Xinjiang, China	W	10	43.68	H1(1), H2(9)	0.200 ± 0.154	0.149 ± 0.089
XH	Inner Mongolia, China	W	25	43.78	H1(18), H5(7)	0.420 ± 0.082	0.290 ± 0.153
TL	Inner Mongolia, China	W	10	43.58	H3(7), H4(3)	0.467 ± 0.132	0.013 ± 0.014
MN	Xinjiang, China	W	20	43.35	H9(20)	0.000	0.000
NL	Xinjiang, China	W	22	43.25	H1(22)	0.000	0.000
ZL	Inner Mongolia, China	W	12	42.96	H5(12)	0.000	0.000
ZW	Liaoning, China	W	16	42.66	H3(11), H4(3), H8(2)*	0.508 ± 0.126	0.016 ± 0.015
CH	Inner Mongolia, China	W	16	42.26	H3(2), H6(14)*	0.233 ± 0.126	0.007 ± 0.009
SD	Shandong, China	W	19	36.25	H4(2), H7(17)*	0.199 ± 0.112	0.110 ± 0.064
GJ	Tibet, China	W	8	29.88	H10(8)	0.000	0.000
BM	Tibet, China	W	8	29.87	H9(4), H10(4)	0.571 ± 0.095	0.126 ± 0.079
XZ	Tibet, China	W	25	29.68	H9(21), H10(4)	0.280 ± 0.101	0.062 ± 0.039
MK	Tibet, China	W	8	29.58	H5(8)	0.000	0.000
DQ	Yunnan, China	W	15	28.47	H10(1), H12(14)	0.133 ± 0.112	0.052 ± 0.035
DX	Yunnan, China	W	16	28.15	H10(16)	0.000	0.000
DM	Yunnan, China	W	16	27.90	H9(16)	0.000	0.000
XG	Yunnan, China	W	19	27.49	H5(19)	0.000	0.000
XL	Yunnan, China	W	21	27.15	H9(10), H10(11)	0.524 ± 0.036	0.116 ± 0.067
C445	Heilongjiang, China	L	10	50.25	H3(5), H4(5)	0.556 ± 0.075	0.015 ± 0.016
C448	Heilongjiang, China	L	11	48.01	H4(11)	0.000	0.000
C254	Inner Mongolia, China	L	16	43.48	H3(12), H4(1), H9(2), H11(1)*	0.442 ± 0.145	0.136 ± 0.078
C564	Xinjiang, China	L	10	43.37	H9(10)	0.000	0.000
C261	Inner Mongolia, China	L	9	40.42	H5(1), H9(5), H21(1)*, H22(2)*	0.694 ± 0.147	0.095 ± 0.061
C187	Gansu, China	L	11	39.71	H4(4), H9(4), H10(1), 13(1)*, H14(1)	0.782 ± 0.095	0.337 ± 0.186
JinMa1	Shanxi, China	B	11	37.3	H4(2), H9(9)	0.327 ± 0.153	0.190 ± 0.109
C274	Xinjiang, China	L	11	37.16	H9(11)	0.000	0.000
C467	Qinghai, China	L	10	36.43	H9(7), H19(2)*, H20(1)*	0.511 ± 0.164	0.213 ± 0.122
C468	Shandong, China	L	10	36.13	H1(9), H2(1)	0.200 ± 0.154	0.006 ± 0.008
C292	Gansu, China	L	10	36.03	H9(8), H14(1), H17(1)*	0.378 ± 0.181	0.135 ± 0.081
C224	Anhui, China	L	11	31.45	H3(11)	0.000	0.000
C666	Tibet, China	L	10	29.72	H10(8), H18(2)	0.356 ± 0.159	0.069 ± 0.046
C269	Tibet, China	L	8	29.71	H9(4), H10(4)	0.571 ± 0.095	0.126 ± 0.079
C290	Guizhou, China	L	10	26.87	H10(10)	0.000	0.000
C001	Yunnan, China	L	10	25.60	H10(10)	0.000	0.000
C218	Guangxi, China	L	10	24.15	H5(10)	0.000	0.000
YunMa7	Yunnan, China	B	10	23.36	H23(10)*	0.000	0.000
Kompolti	Hungary	B	8		H1(8)	0.000	0.000
Futura75	France	B	10		H1(7), H15(2)*, 16(1)*	0.511 ± 0.164	0.093 ± 0.059
Afghanica	The Netherlands (70% indica, 30% sativa)	B	2		H9(2)		
Dame Blanche	The Netherlands (80% indica, 20% sativa)	B	2		H9(2)		
Purple Kush	USA (http://genome.ccbr.utoronto.ca/cgi-bin/hgGateway)	B	1		H9(1)		
Carmagnola	Italy (https://www.ncbi.nlm.nih.gov/)	B	1		H12(1)		
Dagestani	Russia (https://www.ncbi.nlm.nih.gov/)	B	1		H24(1)*		
Yoruba Nigeria	Nigeria, Africa (https://www.ncbi.nlm.nih.gov/)	B	1		H25(1)*		
Cheungsam	Korea (https://www.ncbi.nlm.nih.gov/)	B	1		H1(1)		
*Humulus scandens*	Liaoning and Anhui, China	O	2				
*Humulus yunnanensis*	Yunnan, China	O	1				
*Humulus lupulus*	Czech (https://www.ncbi.nlm.nih.gov/)	O	1				
*Aphananthe aspera*	China (https://www.ncbi.nlm.nih.gov/)	O	1				

W, wild; L, Landrace (domesticated, locally adapted, traditional variety); B, Breeding (cultivar selected by humans for desirable traits); O, Out group; No., sample size; Hd, haplotype diversity; π, nucleotide diversity; Nh, number of haplotype; ^*^, private haplotypes.

Twenty-five wild populations represented by 430 individuals were collected from 2011 to 2016. These populations covered the only distribution ranges of extant wild *Cannabis* throughout China: Inner Mongolia, Jilin, Liaoning, Shandong, Xinjiang, Tibet, and Yunnan provinces or regions. The population size of wild *Cannabis* is generally ranging from hundreds to several thousand individuals. Healthy leaves were collected in the field and immediately dried with silica gel until DNA extractions. To increase the possibility of detecting variation within each population, individuals growing at least 10 m apart were randomly sampled and in addition, eight to thirty plants were sampled from the edges and the interior of populations, depending on the actual population size. For domesticated populations, 27 cultivars represented by 215 individuals were included. Eighteen cultivars (188 individuals) from China were obtained from the Industrial Crops Research Institute, Yunnan Academy of Agricultural Sciences, and two European hemp cultivars, Kompolti and Futura75, were obtained from Hungary and France, respectively. About 200 seeds from each cultivar were planted and during the flowering stage leaves were sampled for DNA extraction. Additionally, two marijuana materials (named Afghanica and Dame Blanche) from The Netherlands were used, whereas sequence data for another five cultivars (Purple Kush, Carmagnola, Dagestani, Yoruba Nigeria, and Cheungsam) were downloaded from GenBank and The Cannabis Genome Browser website (http://genome.ccbr.utoronto.ca/cgi-bin/hgGateway) (Table 1).

**Figure 1:**
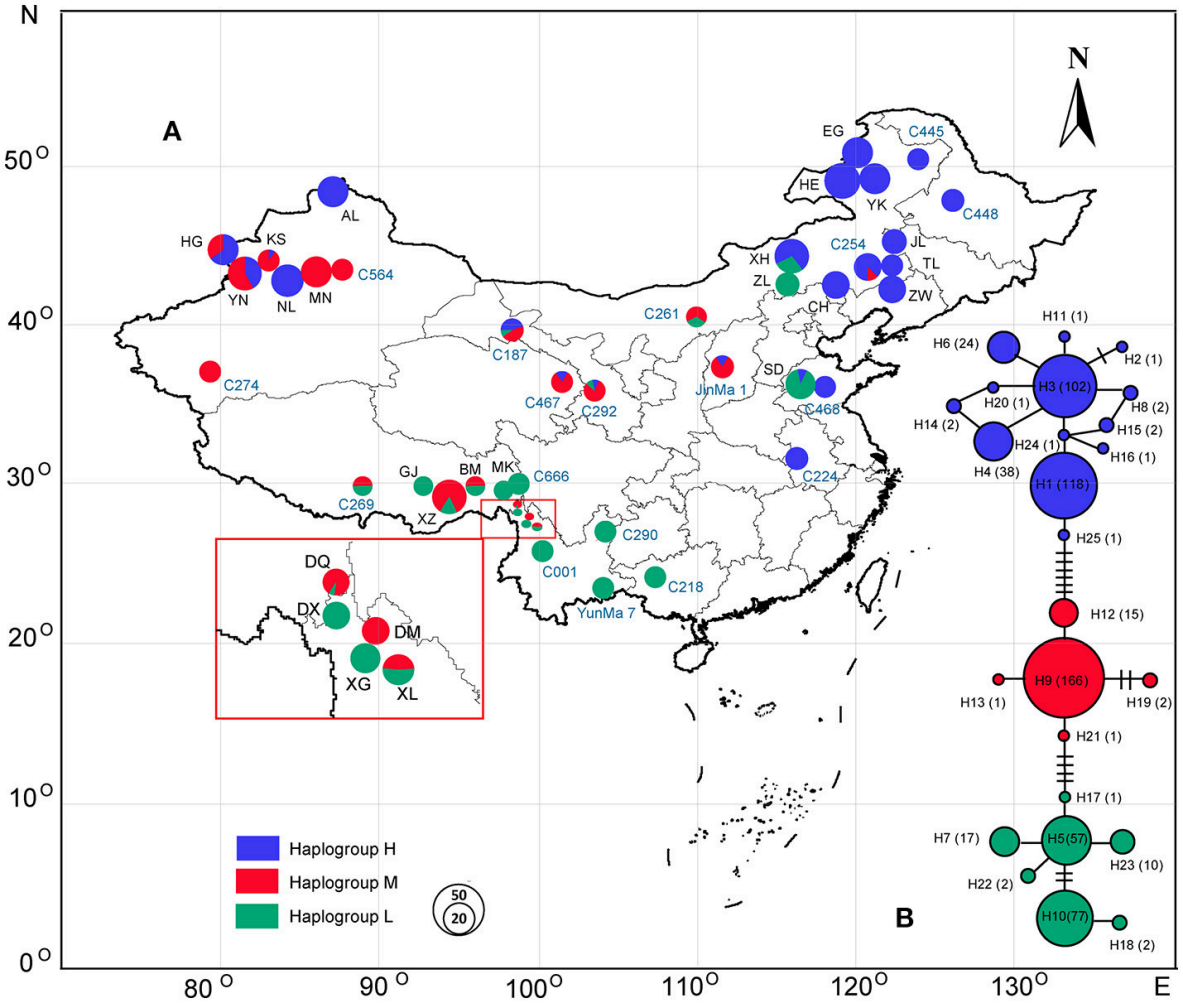
Geographic location of the 43 populations of *Cannabis* analyzed in the present study and haplogroup distribution patterns of *Cannabis* (see Table 1 for population codes); population codes in black represent the wild samples and blue ones are the domesticated accessions. **(B)** The haplotype network generated from the 25 haplotypes of *Cannabis*; pie chart size corresponds to the sample size of each population **(A)** or haplotype **(B)**.

For the 43 hemp populations originating from China [25 wild populations (W) and 18 domesticated cultivars (L and B)] (Table 1, Figure 1), the sampled regions throughout China spanned an area from 50.25° to 23.36° N and from 79.44° to 126.08° E, with an altitude span from about 50 m above sea level in Anhui (C224) to 3,700 m in Tibet (MK).

### DNA Extraction, Primer Development, PCR Amplification, and Sequencing

Total DNA of each sample was extracted from leaf material according to the modified CTAB method (Doyle, 1991; Chen et al., 2015).

To develop genetic markers for population genetic analyses, we first tested 17 universal primer sets, developed for amplification of highly variable chloroplast DNA regions of angiosperms, on six individuals from different wild *Cannabis* populations. However, *Cannabis* individual sequences generated from these primers are too conserved to obtain variable sites suitable for population-level studies despite repeated tests (Zhang et al., 2017). Based on comparisons of the four available whole chloroplast genomes from cultivars of *C. sativa* (Oh et al., 2016; Vergara et al., 2016), we developed five pairs of PCR primers targeting several highly variable chloroplast regions (*rps16; psaI-accD; rps11-rps8; rpl32-trnL; ndhF-rpl32*). These new primers are suitable for the population genetic study of *Cannabis* and its closest relative *Humulus* (Table 2). Due to unsuccessful PCR amplification of the rps16 region of *Humulus* species, a specific forward primer for the genus *Humulus* was designed.

**Table 2:** Primer pairs of cpDNA regions used in this study and polymorphism on the 645 individuals of *Cannabis*.

**cpDNA regions**	**Primers sequence (5' - 3')**	**Annealing temperature (^**°**^C)**	**sequence length (bp)**	**No. of substitutions**	**No. of Indels**	**Total informative characteristics**	***H_***d***_***	***N_***h***_***
*rps16*	rps16^Cf^: TTAAAATAGCAGAGAAAAGATTAT rps16^Hf^: GCAGAGAAAAAAAAGATTCTAATCC rps16^Cr^: AAACGATGTGGTAGAAAGCAAC	58	1081–1084	6	0	6	0.521	7
*psaI-accD*	*psaI* ^Cf^: GAACATGAAGAGATAAAGAAACC *accD* ^Cr^: GCTCCATGCTTTCTCTCCTCCTTTG	55	813–822	5	1	6	0.679	5
*rps11-rps8*	*rps11* ^Cf^:GGGCCTACAGCCATTATGTG *rps8* ^Cr^: CGCTTCCCACATTAGTTAATCCC	55	720	2	0	2	0.614	3
*rpl32-trnL*	*rpl32* ^Cf^: CGACAAATTCTATTAGATAGA *trnL* ^Cr^:: AGAAAATGCCATGCCGCTACTC	53	389–420	3	2	5	0.529	5
*ndhF-rpl32*	*ndhF*: GAAAGGTATKATCCAYGMATATT *rpL32-R*: CCAATATCCCTTYYTTTTCCAA *ndhF* ^Cf^: GGTATAATCCATGAATACTG *rpL32* ^Cr^:: CTGCCCAATATCCTTTCTTTT	47 58	604-620	3	1	4	0.775	6
Total	-	-	3616–3645	19	4	23	0.848	25

*Cf, the forward primer for Cannabis; Cr, the reverse primer for Cannabis; Hf, the reverse primer for Humulus. The four Indels: AAATATT; GAATTGAAAAAAAAA; TATATTAAAA; AAAAAT*.

PCR amplification reactions were carried out in a total volume of 25 μL, containing 2.0 μL DNA template (20–30 ng/μL), 2.5 μL 10 × PCR reaction Buffer (with Mg^2+^), 1.5 μL dNTPs mix (2.5 mmol/L), 0.5 μL each forward and reverse primers (10 μmol/L), 0.3 μL Taq DNA polymerase (5 U/μL, Beijing TransGen Biotech Co., Ltd., China), and 17.7 μL double-distilled water. Amplifications were conducted on an ABI Veriti Thermal Cycler (Applied Biosystems, Foster City, CA, USA) using the following program setting: an initial 4 min pre-denaturation at 94°C, followed by 30 cycles of 45 s at 94°C, 30 s at 47–58°C (Table 2), 45–90 s at 72°C, and a final 10 min at 72°C.

The obtained PCR products were purified with a Gel Extraction and PCR Purification Combo Kit (Beijing Tsingke BioTech Co., Ltd., China) and then bidirectional sequencing was performed on an ABI 3730xl DNA Analyzer (Applied Biosystems, Foster City, CA, USA) employing the same primers used for PCR amplifications. All sequences of the *rps16, psaI-accD, rps11-rps8, rpl32-trnL*, and *ndhF-rpl32* cpDNA regions have been deposited in GenBank under the accession numbers from MG731579 through MG731614.

### Observation of Main Phenological and Morphological Traits

To test whether there are obvious differences among the 43 accessions (including both wild and domesticated germplasms) on phenotypic characteristics, we also carried out a Varieties Evaluation Field Trial in 2016 involving all 43 accessions. The trial site was located in Kunming, Yunnan province of China. This trial was set up as a randomized complete block design with three replicates and each plot was 6 m^2^, with a distance between rows of 40 cm (with a density about 50 plant individuals per square meter). The plots were directly seeded at a depth of 3–5 cm on May 28 in 2016 and all wild-type seeds were pretreated to facilitate germination before sowing about 10 days, and the whole trial was managed with normal management practices. Main phenological and morphological traits for each accession were investigated, including initiation of flowering, full flowering, seed full maturity time, stem diameter, plant height, and number of branches. These data were collected based on 20 individuals randomly selected for each plot (10 individuals for female and male respectively).

### Data Analysis

Raw sequence data of the five amplified DNA fragments (amplicons) were assembled with SeqMan (DNAStar Inc., Madison, WI, USA) and carefully checked for genetic variation together with the chromatograms. Sequences were aligned using the CLUSTAL W (Thompson et al., 1994) followed by manual adjustment implemented in MEGA 6.0 (Tamura et al., 2013). Small insertion/deletion events (indels), excluding long mononucleotide repeats (poly A/T or poly G/C), were counted as single mutations. The haplotypes for each gene marker, and in the combined five-fragment dataset matrix, were identified using DNASP v5.10 (Librado and Rozas, 2009).

Based on the combined five-fragment dataset, the relationships among haplotypes were reconstructed by median-joining (MJ) network method (Bandelt et al., 1999) implemented in the software NETWORK v5.0.0.1 (available at http://www.fluxus-engineering.com) with the maximum parsimony (MP) post-processing option.

To detect genetic diversity and population structure, we carried out the following analyses. The distribution of three haplogroups (identified by phylogenetic tree and network) was plotted on maps of China using ArcGIS v 10.2 (ESRI Inc., Redlands, CA, USA). To define the most differentiated groups of populations we performed a spatial analysis of molecular variance (SAMOVA) using the software SAMOVA v 2.0 (available at http://cmpg.unibe.ch/software/samova2/) based on geographical coordinates and haplotype distribution data of *Cannabis* populations from China. Different hierarchical levels of genetic variation including within populations, among populations within groups and among groups were assessed by the analysis of molecular variance (AMOVA) implemented in Arlequin v 3.5.2.2 (Excoffier and Lischer, 2010), with significance assessed by 1,000 permutations on the 43 populations from China. The 43 populations were grouped into three population groups (Group H, Group M, and Group L) by SAMOVA based on variation in cpDNA or into two morphology groups by morphological and physiological features (the wild Group and domesticated Group) where the population genetic structure and the domestication pattern for *Cannabis* in China were assessed. Indices of nucleotide diversity (π) and haplotype diversity (*Hd*) were calculated for each population, population groups, and for all samples combined, using Arlequin v 3.5.2.2. Also, *Tajima's D* and *Fu's Fs* neutrality tests were conducted. Levels of gene flow (*Fst* and *Nm*) were measured using DNASP v5.10. Mantel tests were conducted to examine the correlation between two matrixes (genetic distances and pairwise geographical distances or latitude differences) with 9,999 permutations using GenALEx v 6.5 (Peakall and Smouse, 2012).

To identify the main climatic factors affecting the distribution of the *Cannabis* genetic lineages, we also tested correlations of 20 bioclimatic factors on a compilation of cpDNA haplogroup frequencies for 43 populations. The values of 19 BioClim variables were extracted by using DIVA-GIS v7.5 (http://www.diva-gis.org/) based on the global climate layer data (at 2.5 arc-min resolution) downloaded from the WorldClim v2.0 database (http://www.worldclim.org/), and the mean day length of cannabis growth season (from the Spring Equinox to Autumnal equinox) were calculated according to solar geometry (Spitters et al., 1986; Yuan et al., 2014) for 43 sampling sites. The correlation between environmental variables and haplogroup frequencies was analyzed by redundancy analysis (RDA). We first assessed the effects of all 20 climatic factors on haplogroup frequencies distribution. And then, to identify a minimum subset of climatic variables that significantly explain variation of genotype spatial distribution, we further tested the multicollinearity in the whole data set, and the redundant factors (variance inflation factors, VIF > 10) were excluded through stepwise regression. To explore the percent variance uniquely explained by each factor, the Analysis of Variance (ANOVA) was calculated. RDA and ANOVA analyses were performed using the vegan package in R version 3.3.1 (R Core Team, 2015).

**Figure 2:**
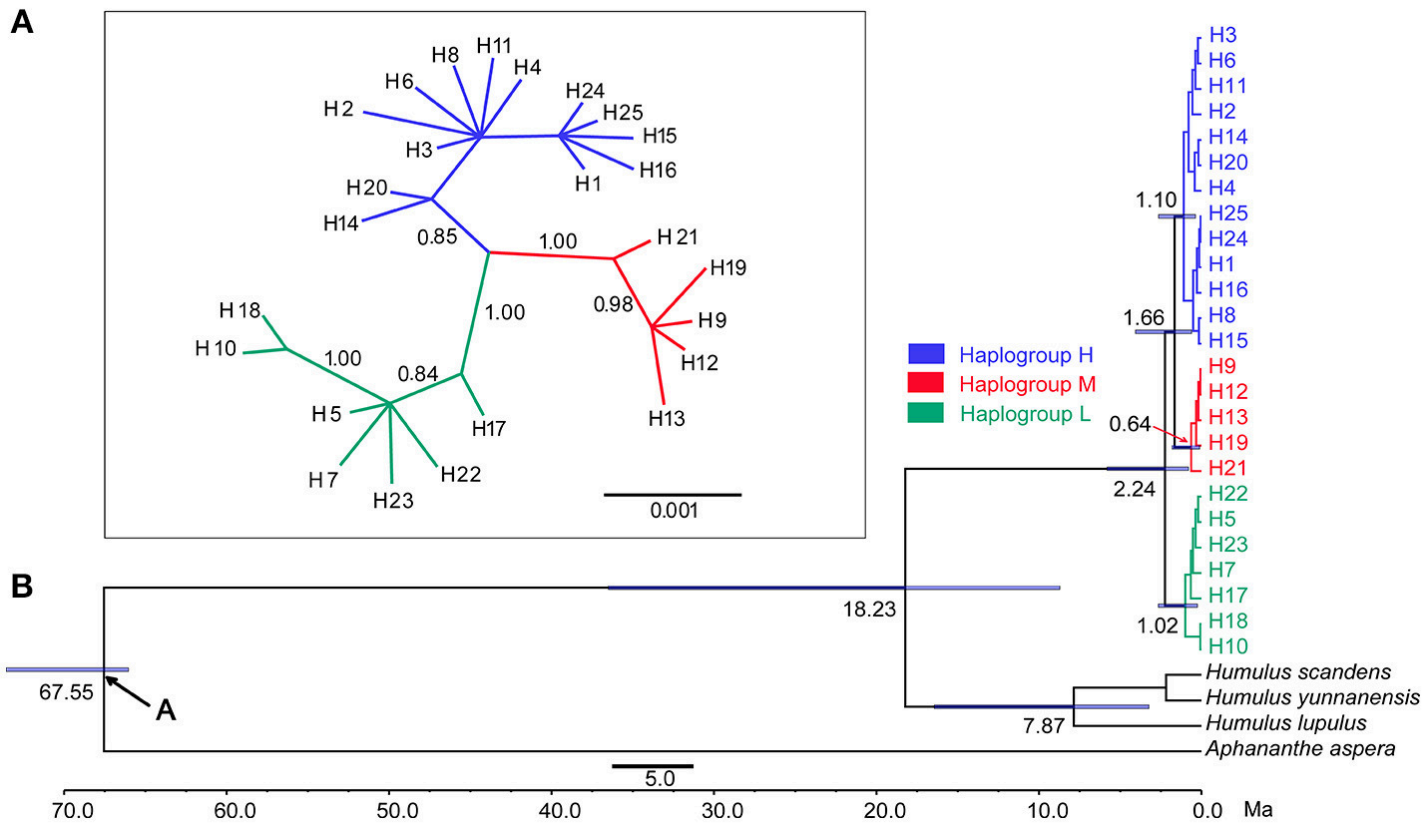
**(A)** Bayesian phylogenetic tree based on cpDNA data. **(B)** Divergence time estimated for the major clades of *Cannabis* by the BAEST analysis (Blue bars indicate the 95% highest posterior density credibility for node ages).

Phylogenetic relationships based on the cpDNA haplotypes were deduced using MrBayes ver. 3.1.2 (Ronquist and Huelsenbeck, 2003). Using the sequences from *Aphananthe* and *Humulus* species as out groups, the divergence times for the major groups of these haplotypes were further estimated with BEAST v1.8.1 (Drummond and Rambaut, 2007) with GTR + G selected by MrModeltest 2.3 as the best substitution model for the data set (Nylander, 2004). The data was analyzed using a relaxed log-normal clock model and a Yule Process speciation model for the tree priors. As the earliest fossil species of *Aphananthe* was reported around 66–72.1 million years ago (Ma) from the Maastrichtian (66–72.1 Ma) in late Cretaceous (Ervín et al., 1986), the stem age of the *Aphananthe* was set to 66 Ma based on the low boundary age (node A in Figure 2B). Prior settings for calibrating node were: offset of 66 Ma, a log mean of 1.0 (log stdev of 0.5). Two independent runs were conducted for 10 million generations. Log files resulted from the two runs were combined using LogCombiner after the first 25% were discarded as burn-ins, and the convergence of the chains was checked in Tracer v1.6 (Rambaut et al., 2014). Similarly, the resulted trees were combined in LogCombiner, and the maximum clade credibility (MCC) tree was produced with Tree Annotator, and then viewed in FigTree v1.4.2.

## Results

### Sequence Characteristics and Identification of cpDNA Haplotypes

We successfully obtained high quality sequences for all the five target cpDNA genes (*rps16, psaI-accD, rps11-rps8, rpl32-trnL, ndhF-rpl32*) for each of the 640 *Cannabis* individual plants. Five additional sequences of *Cannabis* lines were retrieved from the published chloroplast genomes (Table 1). In total, the combined alignment of the five cpDNA fragments (five-gene matrix) covered 3,635 base pairs in length, and harbored 19 single nucleotide polymorphisms (SNPs) and four indels varying up to 38 bp in length (Table 2), thus the proportion of variable sites was 1.57%. The indel mutations introduced by long mononucleotide repeats (poly A/T or poly G/C) were excluded from the analysis. The AT content was 70.5% and a total of 25 haplotypes (H1–H25) were identified based on the genetic variation found among the 645 samples. For the *rps16* intron, and four intergenic spacers (*psaI-accD, rps11-rps8, rpl32-trnL*, and *ndhF-rpl32*), the sequence length and polymorphic informative characters are shown in Table 2.

### Distribution of cpDNA Haplotypes, Phenotypic Characteristics and Genetic Diversity

In the haplotype network (Figure 1B), the 25 haplotypes were split into three distinct haplogroups: Haplogroup H (blue colored), Haplogroup M (red colored), and Haplogroup L (Green colored). Haplogroup H contained 13 haplotypes (H1, H2, H3, H4, H6, H8, H11, H14, H15, H16, H20, H24, H25), Haplogroup M contained 5 haplotypes (H9, H12, H13, H19, H21), and Haplogroup L contained 7 haplotypes (H5, H7, H10, H17, H18, H22, H23). The phylogenetic tree (Figure 2A) also exposed three well-supported lineages corresponding to the above-mentioned three haplogroups illustrated by the haplotype network. The haplotypes are not evenly distributed for each haplogroup (Figure 1B): In Haplogroup H, the two most common haplotypes, H1 (40.1%) and H3 (34.7%), were observed in 15 out of the 20 sampled populations north of 40° N. For Haplogroup M, the most common haplotype H9 (89.7%) and other 4 rare haplotypes were found in the area ranging from 27° to 43° N. In Haplogroup L, seven haplotypes, including the two major haplotypes H5 (34.3%) and H10 (46.4%), were mainly distributed throughout the area south of 30° N.

As the lineages displayed distinct structure by network analyses (Figure 1B) and structural phylogeographic distribution patterns (Figure 1A), SAMOVA analysis (based on a simulated annealing method) was performed to define groups of populations. The result showed that when *k* = 3 the differences between groups (*F*_CT_ = 0.64) was the highest, and the 43 populations from China were divided into three groups: Group H, Group M, and Group L (Figure 1A). Group H included 16 populations mainly from the high latitude region: EG, HE, YK, JL, AL, HG, NL, XH, TL, ZW, CH, C445, C448, C254, C468, and C224. This group largely corresponds to haplogroup H. Group M also included 16 populations but from the middle latitude region: YN, KS, MN, BM, XZ, DQ, DM, XL, C564, C261, C187, JinMa 1, C274, C467, C269, and C292, corresponding to above haplogroup M. Group L included 11 populations mainly from low latitude region: GJ, MK, DX, XG, C290, C001, C218, C666, ZL, SD, and YunMa 7, corresponding to haplogroup L. Frequencies of the three lineages in each population and their geographical distribution are displayed in Figure 1.

Interestingly, we also noted that the main phenotypic traits of 43 wild or domesticated accessions originating from different latitudes shifted along latitudinal gradients (23.36–50.21° N), which matched the regular distribution of three lineages. Our phenotype data (Table S1) indicated there were very high variations among 43 accessions. The six measured traits involved three phenological Characteristics (initiation of flowering, full flowering, and seed full maturity time) and three morphological features (stem diameter, plant height, and number of branches). The correlations between the phenotypes and latitude were assessed, and all six traits had a negative, very strong, and significant (*p* < 0.001) relationship with latitude of origin, Pearson' correlation coefficients (r) respectively were 0.858, 0.949, 0.906, 0.911, 0.914, 0.815 for initiation of flowering, full flowering, seed full maturity, stem diameter, plant height, and number of branches, respectively. When the phenotype of three genetic groups (above mentioned SAMOVA grouping) were compared, group H had the shortest growth time (mean seed-maturity time, 77.2 ± 18.1 days), thinnest stem diameter (0.54 ± 0.22 cm), shortest plant height (99.2 ± 52.4 cm), and fewest branches (3.2 ± 1.7), while Group L had the longest growth time (mean seed-maturity time,133.6 ± 36.8 days), widest stem diameter (1.14 ± 0.40 cm), tallest height (238.0 ± 86.5 cm), and most branches (11.0 ± 3.9), and the traits data of Group M were in-between.

For genetic diversity features, our studies showed that the number of haplotypes is different among the 43 Chinese populations, plus the cultivars Futura75 and Kompolti, ranging from 1 to 5 haplotypes. We observed that out of the 25 haplotypes, 15 private haplotypes were exclusively found in three wild populations (ZW, CH, SD) and in nine cultivated accessions (C254, C261, C187, C292, C467, YunMa7, Futura75, Dagestani, Yoruba Nigeria). Haplotype diversity (*Hd*) and nucleotide diversity (Π) of each population are summarized in Table 1. The domesticated population C187 possessed the highest haplotype diversity (*Hd* = 0.782) and nucleotide diversity (Π = 0.00337), while the lowest number of haplotypes (*Nh* = 1; *Hd* = 0; Π = 0) were found in 18 other populations, including domesticated accessions and wild populations. Among the wild populations, the BM population had the highest haplotype diversity (*Hd* = 0.571), YN population had the highest nucleotide diversity (Π = 0.00379), and ZW had the highest number of haplotypes (*Nh* = 3, *Hd* = 0.508).

## Isolation by Distance and Climatic Correlates of cpDNA Lineages Frequency

To examine whether the observed genetic distributions are correlated to geographical localization, Mantel tests were performed. Between Nei's pairwise genetic distances (Nei, 1978) and the two-dimensional geographical distances (based on longitudinal and latitudinal coordinates), and the results showed that there is a significant positive correlation among the 43 sampled populations from China (*r* = 0.379, *p* = 0.000) and the “isolation-by-distance” pattern was detected. Furthermore, the testing between Nei's pairwise genetic distances and the latitudinal differentiation also showed a significant positive correlation (*r* = 0.348, *p* = 0.000). Similarly, for the 25 wild populations alone, significant positive correlations were found between the genetic distances and pairwise geographical distances (*r* = 0.368, *p* = 0.000), as well as between the genetic distances and latitude differences (*r* = 0.416, *p* = 0.000).

Haplogroup distribution frequencies shifted smoothly along latitudinal gradients and the three lineages distinctively show a high-middle-low latitude distribution pattern (Figure 1). Based on the RDA analysis and ANOVA partition (Table 3), 15 out of the 20 tested BioClim variables had a significant (*p* < 0.05) relationship with haplogroup distribution frequencies for all the 43 populations (Table S2). This result indicated that climate obviously affected the genetic distribution of *Cannabis* populations. When the redundancy factors were removed, only MDL (Mean day length), Bio2 (Mean diurnal range), Bio8 (Mean temperature of wettest quarter), Bio13 (Precipitation of wettest month), Bio14 (Precipitation of driest month), Bio15 (Precipitation seasonality) formed a minimum subset of climatic variables. Based on the ANOVA analysis, MDL was the most significant factor influencing the haplogroup distribution frequencies (*r*^2^ = 0.6024, *p* < 0.001), and the subset of 6 climatic variables totally explained 74.2% of variation, and MDL accounted for the largest fraction of the total explained variation (20.8%).

**Table 3:** ANOVA analyses between BioClim variables and the three cpDNA haplogroup frequencies for 43 Cannabis populations.

**Variables**	**Full name**	**Df**	**Variance**	**F**	**Pr (>F)**
MDL	Mean day length (Spring Equinox-Autumnal eq uinox)	1	0.208027	29.0255	0.001^***^
bio2	Mean diurnal range [mean of monthly (max temp–min temp)]	1	0.009598	1.3391	0.256
bio8	Mean temperature of wettest quarter	1	0.051886	7.2395	0.002^**^
bio13	Precipitation of wettest month	1	0.043932	6.1297	0.004^**^
bio14	Precipitation of driest month	1	0.002271	0.3169	0.756
bio15	Precipitation seasonality (coefficient of variation)	1	0.00323	0.4506	0.64
Residual		36	0.258014		

(^*^p < 0.05;

** p < 0.01;

*** *p < 0.001)*.

### Genetic Structure and Gene Flow

Based on the groups defined by SAMOVA, the analysis of molecular variance (AMOVA) revealed that most variance (69.48%) of the total observed genetic variations was due to variations between-groups, 14.43% was attributed to variance among populations within groups, and 16.10% to variance within the same population (Table 4). F-statistics of all the three levels of hierarchy were highly significant (*p* < 0.001). Population genetic differences (*Fst*) within the High-latitude lineage (Group H) was higher than that of the lower-latitude lineages (Group M and Group L), while gene flow (*Nm*) within Group M and Group L was higher than in Group H (Table 5). For genetic diversity within each group, Group H had the highest haplotype diversity, and Group M had the highest nucleotide diversity and number of haplotypes.

**Table 4:** Analysis of molecular variance (AMOVA) for on the *Cannabis* populations from China based on the five cpDNA regions.

**Source of variation**	**d.f**.	**Sum of squares**	**Variance components**	**Percentage of variation**	**Fixation index (*Fst*)**
**AMONG 3 GROUPS DEFINED BY SAMOVA**					
Among groups	2	2497.697	6.18572	69.48	0.69478^***^
Among populations within groups	40	789.902	1.28440	14.43	0.47264^***^
Within populations	575	824.019	1.43308	16.10	0.83904^***^
Total	617	4111.618	8.90320		
**AMONG 2 GROUPS (WILD & DOMESTICATED)**					
Among groups	1	113.560	0.16155	2.34	0.02345^n.s.^
Among populations within groups	41	3174.039	5.29484	76.85	0.78700^***^
Within populations	575	3174.039	1.43308	20.80	0.79199^***^
Total	617	4111.618	6.88947		
**AMONG 43 POPULATIONS**					
Among populations	42	3287.599	5.36510*Va*	78.92	
Within populations	575	824.019	1.43308*Vb*	21.08	0.78920^***^
Total	617	4111.618	6.79817		

n.s., not significant; ^***^, p < 0.001.

**Table 5:** Population genetic statistics among *Cannabis* population groups based on SAMOVA grouping, the two morphology groups (wild & domesticated) and all samples from China.

***Groups***	***Np***	***Ns***	***Nh***	***Hd***	**π (×10^**−2**^)**	***D***	***Fs***	***Nm***	***Fst***
*Group H*	16	267	9	0.716 ± 0.016	0.159 ± 0.084	−1.013(*n*.*s*.)	10.109(*n*.*s*.)	0.32	0.607
*Group M*	16	219	13	0.500 ± 0.039	0.180 ± 0.094	0.729(*n*.*s*.)	6.390(*n*.*s*.)	1.20	0.294
*Group L*	11	132	6	0.690 ± 0.022	0.059 ± 0.036	−0.007(*n*.*s*.)	2.767(*n*.*s*.)	1.52	0.247
*Group W*	25	430	11	0.838 ± 0.008	0.379 ± 0.189	1.392(*n*.*s*.)	32.064(*n*.*s*.)	0.11	0.820
*Group D*	18	188	15	0.810 ± 0.015	0.311 ± 0.157	1.813(*n*.*s*.)	11.904(*n*.*s*.)	0.15	0.768
*Total*	43	618	21	0.848 ± 0.006	0.367 ± 0.183	1.103(*n*.*s*.)	18.956(*n*.*s*.)	0.12	0.802

*Np, number of populations; Ns, sample size; Nh, number of haplotype; Hd, haplotype diversity; π, nucleotide diversity; D, Tajima's D; Fs, Fu's Fs; Nm, number of effective migrants; Fst, fixation index; n.s., not significant (p > 0.05)*.

When two morphological groups (wild and domesticated) were considered for the same 43 populations, AMOVA analysis indicated low and non-significant (2.34% of molecular variance, *Fst* = 0.023, *p* = 0.19) genetic differentiation between the two groups. Most variance components were present among populations within groups. The degree of population differentiation was slightly higher in the wild group compared to the domesticated group. Results of the neutrality tests for each group and total sample set are shown in Table 5. All values of *Fu's Fs* and *Tajima's D* were statistically non-significance, suggesting stable populations on a different level.

### Divergence Time Estimations

The phylogenetic tree (Figure 2) inferred from the five-gene matrix clustered the 25 haplotypes into a monophyletic clade, in which the haplotypes from the high, middle, and low latitude regions formed three monophyletic subclades, with strong statistical support. The stem age of *Cannabis* (Figure 2B) was estimated at 18.23 Ma with 95% highest posterior density (HPD) 8.83–36.56 Ma, and the crown age of this species was 2.24 Ma, with 95% HPD 0.81–5.81 Ma.

## Discussion

### Distinct Pattern of Lineage Distribution and Genetic Structure

One major finding of this study is that *Cannabis* can be divided into three distinct genetic lineages (Figure 1), namely the H, M, and L haplogroups. Interestingly the haplogroups exhibited latitudinal gradients distribution and this distinctive high-middle-low latitude pattern was supported by NETWORK, AMOVA, SAMOVA, and Mantel Tests based on cpDNA data. High-latitude group members (group H) were mainly distributed in regions north of about 40° N and Low-latitude group members (group L) were mainly distributed in areas south of about 30° N, while the middle-latitude group members (group M) were mainly distributed in the zone between about 30° N and 40° N. This current distribution pattern implies an adaptation to distinct latitudinal gradient climatic features. In the present study, the lineage distribution was significantly correlated with latitude and climatic factors. In particular, the day-length has a strong and significant (*r*^2^ = 0.6024, *p* < 0.001) influence on the haplogroup distribution frequencies in each population by RDA analysis and ANOVA partition (Table 3). Furthermore, our field phenotype trial results showed that phenological and morphological traits had a negative, very strong, and significant correlation with latitude of accession origin. For instance, Group H is characterized by short plant height, thin stem, fewer branches, and short life cycle. On the contrary, Group L demonstrates opposite characteristics compared with Group H. This is well-linked to the quantitative (facultative) short-day plant trait of *Cannabis*. The flowering of *Cannabis* is normally induced by a required duration of days with a minimum uninterrupted period of darkness (10–12 h for most cultivars) (Small, 2015). Due to the sensitivity to photoperiod, shortening day length can promote *Cannabis* plant pre-flowering. On the contrary, prolonged day length would delay this crop from shifting from a vegetative stage into a reproductive stage. Indeed, the northernmost distribution of group L is located at about 43° N, which is consistent with previous observations that cultivars from the southern (low latitude) areas have extended vegetative cycles and failed to produce seeds when grown in the North (High latitude areas) (Pahkala et al., 2008; Amaducci et al., 2012; Small, 2015). Our results suggest that photoperiod sensitivity is a potential factor that prevents group L from extending further north. In contrast, the southernmost boundary of group H is 31° N (landrace C224 in Figure 1A). It was surprising to observe that *Cannabis* lineages still present a distinctive high-middle-low latitude distribution pattern after several thousand years despite human activities. Nevertheless, each of the three haplogroups is not strictly limited to its main corresponding geographical locations: North of 40° N (Haplogroup H), 30 to 40° N (Haplogroup H), and South of 30° N (Haplogroup L). Some haplotypes of the haplogroups were aberrantly growing out of the main distribution latitude range (Figure 1A). For instance, haplotype H3 in cultivar C224, which belongs to Haplogroup H, was found in lower latitude areas around 31° N; while the haplotype H5 in wild population XH and ZL, which belongs to Haplogroup L, was found at a higher latitude area around 43° N. These exceptions may result from the influences of human agricultural activities. Clarke and Merlin (2016) have stated, “Humans and the Cannabis plant share an intimate history spanning millennia.” There might have been much more stringent distribution limits between haplogroups prior to human activities (see below).

The high genetic diversity of this crop has been reported based on nuclear genetic markers (Gao et al., 2014; Sawler et al., 2015; Soler et al., 2017), but this is the first report of genetic diversity from cpDNA markers. The rather low mutation rate among numerous organelle loci of *Cannabis* (Gilmore et al., 2007; Zhang et al., 2017), makes genetic analyses of populations based on single organelle sequence extremely difficult. Our results revealed a high level of haplotype diversity (*Hd* = 0.848) at the species level, a strong genetic differentiation among the three groups (*Fst* = 0.695), and the molecular variations observed are mostly between-cultivars (76.85%) or among groups (69.48%). It is worth noting that genetic variation at different levels of hierarchy contrasts to previous studies based on nuclear markers (Gilmore et al., 2003; Chen et al., 2015; Soler et al., 2017), where the largest molecular variation observed was due to differences within cultivars, instead of among cultivars. These contrasting results are probably due to the fact that the cpDNA markers are maternally inherited, and detect therefore variations only from the maternal parent, instead of an unspecified mixture of both parents, which occurs for nuclear markers. The haploid and non-recombining nature of the cpDNA makes it possible to better trace genealogical histories in plant populations (Avise, 2009).

### Three Subspecies Classification

The genus *Cannabis* was previously placed in family *Moraceae*, then in its own family *Cannabaceae* together with *Humulus* (Rendle, 1925). This family contains ten genera based on molecular phylogenies (Sytsma et al., 2002; Mabberley, 2008; Yang et al., 2013). The cultivation and selection of hemp has been performed for several thousand years, and this has resulted in difficulty when classifying *Cannabis* accessions based only on morphological traits. In recent studies, three lineages have been identified in *Cannabis* by enzyme variants analysis (Hillig, 2005), 7 polymorphic sites of organelle DNA sequences (Gilmore et al., 2007), and EST-SSR markers (Gao et al., 2014) based on worldwide sampling. However, whether these three lineages should be treated as three distinct species, three varieties of a single species or other taxonomic treatments have been debated (Hillig, 2005; Gilmore et al., 2007; Piluzza et al., 2013; Small, 2015; Mcpartland and Hegman, 2018). In the present study, 645 *Cannabis* individuals (all 43 populations from China and 9 accessions from the other countries or regions) were split into three gene pools without exception. On the phylogenetic tree, all *Cannabis* haplotypes formed a monophyletic clade (Figure 2B) containing three distinct subclades, with each subclade significantly different from the others (Figure 2A). At first glance, the three distinct subclades could be treated as three different species corresponding to the three commonly recognized species *C. sativa, C. indica*, and *C. ruderalis*. However, there is no reproductive isolation that exists between these lineages in nature based on our observations as well as recognitions by most researchers (Beutler and Marderosian, 1978). Furthermore, few sequence variations have been detected in *Cannabis* chloroplast DNA: < 0.03% for the whole chloroplast genomes based on four *Cannabis* cultivars and < 0.24% for the 16 cpDNA non-coding regions based on six individuals of wild *Cannabis* (Zhang et al., 2017); < 0.1% for the 7 cpDNA regions (Gilmore et al., 2007). In addition, significantly lower divergence (0.41%) was observed between materials identified as *C. sativa* and *C. indica* based on DNA barcoding sequences (*rbcL, matK, trnH-psbA, trnL-trnF, ITS*), compared to the mean divergence of 3.0% that separated five pairs of plants considered as different species such as *Humulus lupulus* and *H. japonicus* in *Canabaceae* (McPartland and Guy (2014). These accumulating pieces of evidence also hint that a rank below that of species is more reasonable. Thus we suggest that *Cannabis* should be considered as a monotypic genus with only one species, *Cannabis sativa* L. Considering that the three distinctive lineages revealed by cpDNA molecular markers also clearly demonstrated obvious geographic regions as stated above, this species can be further divided into three subspecies. Meanwhile, based on nomenclature history of this species, original geographic range, and basic difference in phenotype, we recommend the naming of the three subspecies as: *Cannabis sativa* subsp. *sativa, C. sativa* subsp. *indica*, and *C. sativa* subsp. *ruderalis*, corresponding to the Haplogroup M, Haplogroup L, and Haplogroup H, respectively. Small and Cronquist (1976) also pointed out that *C. sativa* subsp. *sativa* is typically distributed at areas with latitudes north of 30° N. Our present results that the haplogroup M (i.e., subsp. *sativa*) is distributed in areas ranging from 27 to 43° N, is largely consistent with the observations by Small and Cronquist.

### Divergence Time Inference and Evolutionary History

In the present study, we included *Aphananthe aspera*, the basal taxon of the family *Cannabaceae*, and all the three *Humulus* species (the sister group of *Cannabis*) as outgroups for the dating analysis based on cpDNA markers and large numbers of *Cannabis* individuals. The reconstructed phylogenetic tree (Figure 2) shows the stem age of *C. sativa* is at 18.23 Ma (95% HPD: 8.84–36.6 Ma), which means *Humulus* and *Cannabis* diverged from a common ancestor before 18.23 Ma. This time period is in agreement with the divergence time (about 14 Ma) inferred by Zhang et al. (2017). In fact, the history of *Cannabis, Humulus* and their extinct sister genus can be dated back to the Oligocene and Miocene Epoch (33.9–5.33 Ma) according to the fossil records (Tiffney, 1986; McPartland, 2018). The crown age of *C. sativa* is at 2.24 Ma (95% HPD: 0.81–5.81 Ma), which is also the stem age of the three lineages. This diversification time coincides with the Quaternary glaciation, the last of five known glaciations during Earth's history which is thought to have started at 2.58 Ma, indicating that the Quaternary glaciation could have played a major role in the evolutionary history of the three subspecies of *C. sativa*. The current distribution of the three subspecies could be explained as a consequence of secondary contact after historical divergence events.

The Central-Asia-Origin has been the prevalent opinion for *C. saltiva* (de Candolle, 1885; McPartland, 2018), although some botanists considered Europe as the center of origin (Thiébaut de Berneaud, 1835; Keppen, 1886), or a region spanning Asia and Europe (Herder, 1892; Vavilov, 1926). However, our molecular analyses revealed for the first time that the low latitude region distributed subsp. *indica* (Haplogroup L) possesses the basal group position within *Cannabis*, indicating that this species is possibly originated from low latitude areas in the evolutionary history of this plant. This finding does not support the hypothesis of the Central-Asia-origin of *Cannabis*, but is partly in agreement with the speculation of Linnaeus (1737) that the native range of *C. saltiva* was India Orientali (encompassing the Indian subcontinent, southeastern Asia, and the Malay Archipelago), Japonia (Japan), and Malabaria (the Malabar coast of southwest India). Indeed, the seeds from wild *Cannabis* populations in India are remarkably small, unlike those collected from any other area, also indicating that the wild Indian populations may be an ancient wild form (Small, 2015).

### Multiregional Domestication Origin of *Cannabis* Plant

Each of the three haplogroups (M, L, and H) identified in this study contains haplotypes from both wild populations and cultivars. Within each haplogroup, the wild and domesticated populations shared the most common haplotypes. For instance, haplotype H1, H3, and H4 are the most common haplotypes shared by the wild and domesticated populations in Group H; similar trends are observed for haplotype H9 in Group M, and haplotypes H5 and H10 in the Group L. The fact that the haplotype of the domesticated *Cannabis* cultivars are not limited to one of the three haplogroups indicates that there are probably multiregional domestication origins for this crop from the three subspecies of *Cannabis*. Otherwise, the same genotype (haplogroup) should have been detected in different cultivars from high-middle-low latitude regions if the cultivars were domesticated from one single region. AMOVA analyses results also demonstrate that there is no significant difference (*Fst* = 0.023) between the wild population group and domesticated cultivar group based on cpDNA data. This molecular evidence is in accordance with the multiregional origin of human use of the cannabis plant proposed based on archaeological investigation (Long et al., 2016) and Fossil pollen studies (Mcpartland et al., 2018). Actually, contemporaneous cannabis achenes (5,000–10,200 years ago) have been found in more than ten different archaeological sites located in the two distal parts (both Europe and East Asia) of the continent (Long et al., 2016). Thus the domestication of *C. sativa* could have occurred in more than three areas in Eurasia.

## Author Contributions

QZ designed and performed the research, analyzed the data, and wrote the manuscript. QZ and XC contributed equally as first author. LT and ES carried out pre-experiment research and revised the manuscript in detail. HG, RG, MG, and YX conducted field work. XC and MY provided the technical assistance. MY organized this work. All authors contributed to and approved the final manuscript.

### Conflict of Interest Statement

The authors declare that the research was conducted in the absence of any commercial or financial relationships that could be construed as a potential conflict of interest.
