# Supplementary material for: Latitudinal Adaptation and Genetic Insights Into the Origins of Cannabis sativa L
Source: Front Plant Sci. 2018 Dec 21;9:1876. doi: 10.3389/fpls.2018.01876 (PMC6309158; doi:10.3389/fpls.2018.01876)
Supplement: Supplementary file 1 [file Table_1.docx]

**Table S1**

**The main phenological and morphological characters for each accession**

| **Group**  **(SOMOVA)** | **Accessions/**  **populations** | **Latitude**  **(° N)** | **phenological** | | | **morphological** | | |
| --- | --- | --- | --- | --- | --- | --- | --- | --- |
|  |  |  | **beginning flowering** | **full flowering** | **seed full maturity** | **stem diameter**  **(cm)** | **plant height**  **(cm)** | **number of branches (>15 cm)** |
| Group H | EG | 50.21 | 25 | 41 | 65 | 0.33 | 65.3 | 1.0 |
|  | HE | 49.28 | 25 | 41 | 65 | 0.33 | 58.7 | 2.6 |
|  | YK | 49.25 | 26 | 42 | 69 | 0.38 | 76.9 | 3.8 |
|  | JL | 45.02 | 30 | 54 | 74 | 0.44 | 89.6 | 5.6 |
|  | AL | 48.2 | 30 | 55 | 76 | 0.38 | 73.2 | 2.1 |
|  | HG | 44.21 | 32 | 58 | 76 | 0.58 | 79.7 | 4.2 |
|  | XH | 43.78 | 29 | 45 | 70 | 0.3 | 63.5 | 1.5 |
|  | TL | 43.58 | 29 | 47 | 66 | 0.74 | 91.7 | 3.1 |
|  | NL | 43.25 | 30 | 50 | 76 | 0.44 | 77.8 | 2.7 |
|  | ZW | 42.66 | 31 | 57 | 76 | 0.52 | 103.8 | 6.0 |
|  | CH | 42.26 | 29 | 55 | 73 | 0.7 | 117.7 | 5.8 |
|  | C445 | 50.25 | 25 | 41 | 65 | 0.35 | 62.1 | 0.9 |
|  | C448 | 48.01 | 27 | 46 | 79 | 0.54 | 76.9 | 1.7 |
|  | C254 | 43.48 | 30 | 53 | 71 | 0.71 | 130.9 | 4.4 |
|  | C468 | 36.13 | 41 | 64 | 95 | 0.85 | 149.9 | 1.6 |
|  | C224 | 31.45 | 68 | 92 | 139 | 1.1 | 269.8 | 3.7 |
| Mean±SD  (N=16) |  | range:  50.21-31.45 | 31.7±10.4 | 52.6±12.6 | 77.2±18.1 | 0.54±0.22 | 99.2±52.4 | 3.2±1.7 |
| Mean±SD  (N=14)^a^ |  | range:  50.21-42.66 | 28.4±2.4 | 48.9±6.4 | 71.5±4.9 | 0.48±0.15 | 83.4±21.4 | 3.2±1.8 |
| Group M | YN | 43.84 | 35 | 58 | 76 | 0.5 | 80.5 | 4.8 |
|  | KS | 43.68 | 35 | 61 | 79 | 0.77 | 130.6 | 9.6 |
|  | MN | 43.35 | 32 | 56 | 82 | 0.45 | 86.3 | 4.1 |
|  | BM | 29.87 | 48 | 81 | 115 | 0.87 | 167.9 | 12.5 |
|  | XZ | 29.68 | 48 | 81 | 117 | 0.94 | 176.4 | 13.9 |
|  | DQ | 28.47 | 49 | 81 | 114 | 1.15 | 246.2 | 14.7 |
|  | DM | 27.9 | 50 | 88 | 129 | 1.18 | 261.6 | 18.2 |
|  | XL | 27.15 | 44 | 89 | 124 | 1.1 | 216.4 | 11.3 |
|  | C564 | 43.37 | 30 | 55 | 80 | 0.5 | 84.1 | 4.7 |
|  | C261 | 40.42 | 37 | 65 | 87 | 0.72 | 149.2 | 4.7 |
|  | C187 | 39.71 | 35 | 57 | 89 | 0.62 | 93.8 | 7.4 |
|  | JinMa1 | 37.3 | 36 | 62 | 103 | 0.96 | 199.5 | 3.7 |
|  | C274 | 37.16 | 43 | 68 | 96 | 1.01 | 183.5 | 4.6 |
|  | C467 | 36.43 | 43 | 68 | 92 | 0.95 | 177.5 | 6.3 |
|  | C292 | 36.03 | 43 | 69 | 95 | 0.73 | 205.6 | 4.6 |
|  | C269 | 29.71 | 48 | 81 | 115 | 0.96 | 188.6 | 6.2 |
| Mean±SD  (N=16) |  | range:  43.84-27.15 | 41.0±6.7 | 70.1±11.8 | 99.6±17.3 | 0.84±0.23 | 165.5±57.1 | 8.2±4.6 |
| Mean±SD  (N=12)^b^ |  | range:  40.42-27.15 | 43.7±5.3 | 74.2±10.6 | 106.3±144.4 | 0.93±0.17 | 188.9±43.8 | 9.0±4.9 |
| Group L | ZL | 42.96 | 29 | 47 | 62 | 0.32 | 58.8 | 3.2 |
|  | SD | 36.25 | 45 | 76 | 111 | 0.87 | 180.6 | 7.5 |
|  | GJ | 29.88 | 50 | 85 | 120 | 1.1 | 177.1 | 12.1 |
|  | MK | 29.58 | 48 | 81 | 115 | 0.88 | 181.2 | 13.4 |
|  | DX | 28.15 | 47 | 81 | 124 | 1 | 209.1 | 15.1 |
|  | XG | 27.49 | 53 | 101 | 145 | 1.29 | 285 | 14.1 |
|  | C666 | 29.72 | 48 | 83 | 133 | 1.05 | 271.9 | 6.8 |
|  | C290 | 26.87 | 64 | 82 | 116 | 1.11 | 251.6 | 10.4 |
|  | C001 | 25.6 | 85 | 104 | 180 | 1.6 | 342.7 | 9.3 |
|  | C218 | 24.15 | 83 | 102 | 179 | 1.68 | 324.8 | 15.2 |
|  | YunMa7 | 23.36 | 92 | 117 | 185 | 1.59 | 335.6 | 13.7 |
| Mean±SD  (N=11) |  | range:  42.96-23.36 | 58.5±19.9 | 87.2±18.6 | 133.6±36.8 | 1.14±0.40 | 238.0±86.5 | 11.0±3.9 |
| Mean±SD  (N=9)^c^ |  | range:  29.88-23.36 | 63.3±18.4 | 92.9±13.3 | 144.4±29.4 | 1.26±0.30 | 264.3±64.3 | 12.2±2.9 |

N, the number of accessions or populations;  ^a^,except C468 and C224; ^b^,except YN, KS, MN and C564; ^c^, except ZL and SD; phonological characteristics, the days from seeding to flowering or seed maturity.

**Table S2**

**Correlation coefficients (r^2^) and levels of significance between the 20 BioClim variables and the three cpDNA haplogroup frequencies for 43 *Cannabis* populations.**

| Code | Full name | r^2^ | Pr(>r) |
| --- | --- | --- | --- |
| MDL | Mean day length (Spring Equinox - Autumnal eq uinox) | 0.6024 | 0.001 *** |
| bio1 | Annual mean temperature | 0.1852 | 0.019 * |
| bio2 | Mean diurnal range (mean of monthly (max temp–min temp)) | 0.0585 | 0.303 |
| bio3 | Isothermality (bio2/bio7) (× 100) | 0.5478 | 0.001 *** |
| bio4 | Temperature seasonality (standard deviation × 100) | 0.6253 | 0.001 *** |
| bio5 | Max temperature of warmest month | 0.2373 | 0.005 ** |
| bio6 | Min temperature of coldest month | 0.3962 | 0.001 *** |
| bio7 | Temperature annual range (bio5-bio6) | 0.5869 | 0.001 *** |
| bio8 | Mean temperature of wettest quarter | 0.2356 | 0.006 ** |
| bio9 | Mean temperature of driest quarter | 0.3801 | 0.001 *** |
| bio10 | Mean temperature of warmest quarter | 0.1828 | 0.021 * |
| bio11 | Mean temperature of coldest quarter | 0.4119 | 0.001 *** |
| bio12 | Annual precipitation | 0.3089 | 0.001 *** |
| bio13 | Precipitation of wettest month | 0.3121 | 0.002 ** |
| bio14 | Precipitation of driest month | 0.0669 | 0.278 |
| bio15 | Precipitation seasonality (coefficient of variation) | 0.0421 | 0.443 |
| bio16 | Precipitation of wettest quarter | 0.3491 | 0.001 *** |
| bio17 | Precipitation of driest quarter | 0.059 | 0.334 |
| bio18 | Precipitation of warmest quarter | 0.3518 | 0.001 *** |
| bio19 | Precipitation of coldest quarter | 0.0606 | 0.32 |

(*p < 0.05; **p < 0.01; ***p < 0.001).
